# Toward Population-Based Genetic Screening for Hereditary Amyloidosis∗

**DOI:** 10.1016/j.jaccao.2021.09.005

**Published:** 2021-10-19

**Authors:** Nosheen Reza, Scott M. Damrauer

**Affiliations:** a Division of Cardiovascular Medicine, Department of Medicine, Perelman School of Medicine at the University of Pennsylvania, Philadelphia, Pennsylvania, USA; b Division of Vascular Surgery and Endovascular Therapy, Department of Surgery, and Department of Genetics, Perelman School of Medicine at the University of Pennsylvania, Philadelphia, Pennsylvania, USA; c Corporal Michael J. Crescenz Veterans Affairs Medical Center, Philadelphia, Pennsylvania, USA

**Keywords:** amyloidosis, cardiomyopathy, electronic health records, genomics

**Figure:**
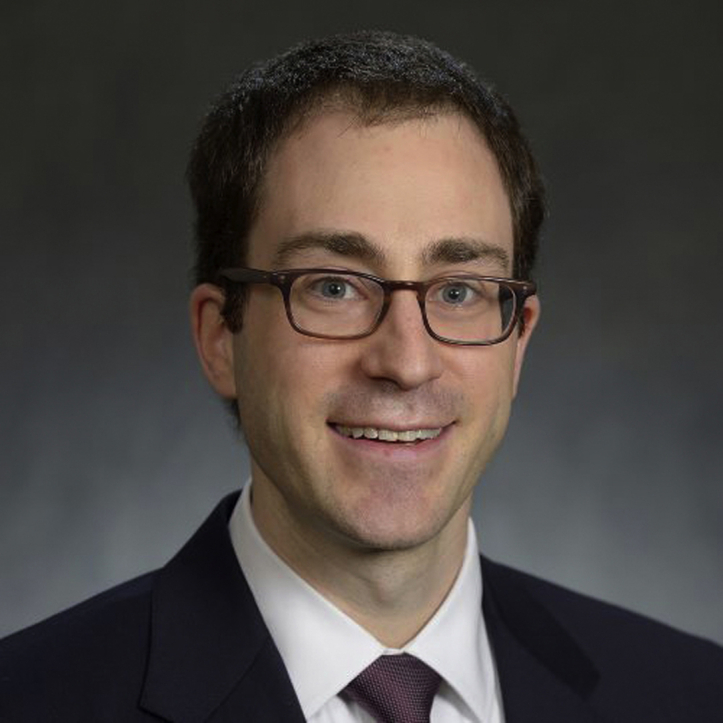

In hereditary amyloidosis (hATTR), genetic variation in the transthyretin gene (*TTR*) leads to destabilization of the TTR protein and results in protein misfolding, misaggregation, and aberrant deposition. Over 130 variants in *TTR*, transmitted via autosomal dominant inheritance, can cause this highly morbid multisystem disease. Cardiomyopathy (CM) and polyneuropathy are recognized as the predominant manifestations of hATTR, although phenotypic penetrance is variable and age-dependent. The considerable ancestral and geographic heterogeneity in the distribution of disease-causing *TTR* genetic variants and historical under-recognition of hATTR have complicated the estimation of true disease prevalence and of genotype-phenotype correlations (1).

Although traditional prospective cohort, registry, and autopsy studies have yielded significant advances in our understanding of hATTR epidemiology and outcomes, hATTR remains underdiagnosed for a number of patient-, physician-, and systems-related reasons, including lack of awareness of the clinical manifestations and of newer treatment options (2). It is in this landscape that genetic biobanks linked to electronic health records (EHR) have emerged as effective tools for the characterization of rare genetic variants. Specifically for hATTR, EHR-linked biobanks have recently been leveraged to identify novel associations between the *TTR* V142I pathogenic variant (p.Val142Ile or p.V142I, historically p.Val122Ile or V122I) and polyneuropathy (3), and between V142I and heart failure among individuals of African or Hispanic/Latino ancestry (4). Moreover, these biobanks have the potential to facilitate the identification of individuals with genetic predisposition to rare diseases before their development of advanced and potentially untreatable disease. For hATTR, disease-modifying therapies such as gene silencers and stabilizers for the often life-limiting manifestation of hATTR-CM are indicated for individuals with New York Heart Association (NYHA) functional class I-III heart failure. Modeling studies have demonstrated longer survival at a better NYHA functional class and gain in quality-adjusted life-years for patients with ATTR-CM on the *TTR* stabilizer tafamidis compared with those treated with standard of care (5). Consequently, strategies to more broadly characterize hATTR disease burden and identify individuals who may benefit from early diagnosis and therapeutic intervention are urgently needed.

In this issue of *JACC: CardioOncology*, Carry et al (6) used the Geisinger MyCode Community Health Initiative—a large, EHR-linked population-based biobank—to evaluate the phenotypic associations of pathogenic and likely pathogenic (P/LP) variants in *TTR* beyond just V142I. The authors hypothesized that this genome-first strategy would reveal cardiac, ophthalmologic, and neurological phenotypes in presymptomatic, at risk individuals and provide further evidence on the utility of population-based genomic screening for hATTR. To accomplish this, they compared cardiac and neurological phenotypes of interest as defined by International Classification of Diseases-9th or -10th Revision diagnosis codes between individuals that did or did not carry *TTR* P/LP variants. Of the 134,753 eligible participants in MyCode, 0.12% (n = 157) individuals carried 1 of 7 total *TTR* P/LP variants present in the biobank. This represents a carrier rate of 1:858, and when extrapolated in a racially matched fashion to the U.S. population at large, represents an estimated carrier rate of 1:222. These rates are significantly higher than the reported prevalence of hATTR in the U.S. population, which has been estimated using phenotype centric approaches to be 1:100,000 (7).

When the data were examined in aggregate, there were no significant differences in the frequency of cardiac, neurological, ophthalmologic, or multisystem phenotypes between the 157 *TTR* P/LP variant carriers and noncarriers, nor were there differences in echocardiographic traits. Because hATTR-CM has been largely reported to present in the seventh decade of life (8) and the median age of participants in this cohort who carried a P/LP *TTR* variant was 52 years, the authors then performed an age-restricted case-control analysis with the hypothesis that the age-dependent penetrance of hATTR mediated the discovery of phenotypic associations in the overall cohort. When limited to the individuals aged 60 years or older (n = 64,316), *TTR* P/LP variant carriers (n = 49) had approximately 2.0- to 3.3-fold higher odds of heart failure, cardiomyopathy, and atrial fibrillation compared with noncarriers but did not exhibit differences in noncardiac phenotypes compared with those without P/LP *TTR* variants.

The authors also performed a subgroup analysis of the 113 participants who carried the V142I variant. These individuals had significantly higher odds of atrial fibrillation, sick sinus syndrome, and hepatomegaly compared with all others in MyCode. Although this analysis was not further stratified by race, the phenotypic association with atrial fibrillation aligns with the recent discovery of a higher risk of atrial fibrillation and ischemic stroke, regardless of the presence of concomitant heart failure, in Black V142I variant carriers in the ARIC (Atherosclerosis Risk In Communities) study (9).

Overall, the work by Carry et al (6) demonstrates the utility of a population-based genome-first screening approach to characterize a rare disease in which an early molecular diagnosis could potentially mitigate its significant morbidity and mortality burden. Even though this study used a population-based biobank of relatively healthy participants predominantly of European ancestry, the estimated hATTR variant carrier prevalence was strikingly higher, at approximately 1:200-900, than what is currently accepted as the manifest disease prevalence of 1:100,000. Although large-scale genetic screening studies of similar unselected populations are needed to validate this finding, it is clear that there is a sizable but unrecognized population at risk for hATTR.

The current study also reinforces the unequivocal underdiagnosis of hATTR-CM. Despite the preponderance of cardiac disease in participants aged 60 years or older, the number of individuals in this group with an EHR diagnosis of cardiac amyloidosis was exceedingly low. Although this finding is not novel, it unfortunately highlights the critical deficiencies in hATTR recognition among clinicians. Furthermore, of the 113 individuals with the *TTR* V142I pathogenic variant, 91 were of African ancestry, and none of these individuals carried an EHR diagnosis of cardiac amyloidosis. Underappreciation and underdiagnosis of hATTR in this population is especially likely to be compounded by systemic inequities in health care access and delivery among historically underserved populations. Although it remains to be seen whether early identification of and intervention for V142I variant carriers yields improved cardiovascular outcomes, these and other similar epidemiological observations support prioritizing prospective studies targeting hATTR screening in diverse populations.

Beyond the search for population prevalence of known hATTR phenotypes as was performed in this work, additional genomic screening applications in large biobanks include phenome-wide association approaches to identify additional predisposing or associated phenotypes that have yet to be correlated with the disease of interest (10). Theoretically, recognizing disease manifestations that may emerge before the cardiomyopathy or polyneuropathy phenotypes may provide the opportunity for earlier intervention. Moreover, other potential influences on hATTR penetrance, such as gene-environment interactions, can be investigated by using social determinants of health data, eg, environmental exposures and lifestyle behaviors, derived from the EHR. The success of advanced data-driven association studies is heavily reliant upon data quality and acquisition, and standardization and validation of evolving EHR-based phenotypes remain challenging.

Given the pressing need to identify the optimal time window for diagnostic and therapeutic intervention for asymptomatic or subclinically affected individuals, Carry et al (6) recapitulate data for the age-dependent penetrance of hATTR. They offer strong supporting evidence for the 50- to 60-year age range as a high yield period for intensification of clinical screening for individuals at risk for hATTR-CM, before their overt development of cardiac disease. With the mounting evidence for *TTR* genotype, especially V142I, as a risk biomarker for cardiomyopathy, heart failure, and atrial fibrillation and as a causal predictor of disproportionate cardiovascular mortality risk for Black Americans, the time to incorporate genetic testing and genotype-first paradigms into our diagnostic cascades has undoubtedly arrived. This genome-first, EHR-linked biobank approach represents an exciting opportunity to make longitudinal, cost-effective, and impactful population-based genomic screening widely accessible—a necessary step toward improving the prognosis of hATTR-CM for all.

## Funding Support and Author Disclosures

Dr Reza is supported by the National Center for Advancing Translational Sciences of the National Institutes of Health under award number KL2TR001879. The content is solely the responsibility of the authors and does not necessarily represent the official views of the National Institutes of Health. Dr Damrauer has received research support to the University of Pennsylvania from RenalytixAI; has received personal consulting fees from Calico Labs, both outside the scope of the current work; and is supported by the U.S. Department of Veterans Affairs (IK2-CX001780). This publication does not represent the views of the Department of Veterans Affairs or the United States Government.
